# Roses Are Red, Socks Are Blue: Switching Dimensions Disrupts Young Children’s Language Comprehension

**DOI:** 10.1371/journal.pone.0158459

**Published:** 2016-06-29

**Authors:** Ron Pomper, Jenny R. Saffran

**Affiliations:** Department of Psychology, University of Wisconsin-Madison, Madison, Wisconsin, United States of America; Birkbeck College, UNITED KINGDOM

## Abstract

Language is used to identify objects in many different ways. An apple can be identified using its name, color, and other attributes. Skilled language comprehension requires listeners to flexibly shift between different dimensions. We asked whether this shifting would be difficult for 3-year-olds, who have relatively immature executive function skills and struggle to switch between dimensions in card sorting tasks. In the current experiment, children first heard a series of sentences identifying objects using a single dimension (either names or colors). In the second half of the experiment, the labeling dimension was switched. Children were significantly less accurate in fixating the correct object following the dimensional switch. This disruption, however, was temporary; recognition accuracy recovered with increased exposure to the new labeling dimension. These findings provide the first evidence that children’s difficulty in shifting between dimensions impacts their ability to comprehend speech. This limitation may affect children’s ability to form rich, multi-dimensional representations when learning new words.

## Introduction

Speakers can identify the same object in many different ways. We can refer to an apple by its name (‘Find the apple’), its color (‘Look at the red one’), an associated action (‘Which one can you eat?’), and its category membership (‘Where’s the fruit?’). Skilled language comprehension involves not only learning to identify an object using multiple dimensions and labels, but also to flexibly shift between these dimensions as the context or conversation demands. This flexibility in language comprehension may be particularly difficult for children, given their relatively immature executive function [1,2]. Executive function (EF), or cognitive control, is the ability to control our attention and actions by overriding dominant or prepotent responses, and consists of a constellation of components including working memory, inhibition, and task switching [3].

Flexibility in skilled language comprehension involves not only switching between dimensions but also switching between competing interpretations. In the sentence “Put the apple on the towel in the box,” the phrase *on the towel* is temporarily ambiguous; it could refer to the destination of the action (i.e., where to put the apple) or serve as a modifier (i.e., which apple). To comprehend structurally-ambiguous sentences, listeners must flexibly shift between competing interpretations as the sentence unfolds. An emerging field of research indicates that this flexibility requires EF [4,5]. Comprehending sentences with structural ambiguity evokes greater activity in regions of the prefrontal cortex (PFC) that are associated with EF [6,7,8]. Adults experience temporary difficulty when comprehending structurally-ambiguous sentences (the garden-path effect [9]). For adults with impairments in EF due to PFC lesions [10] and typically developing children [9], however, the difficulty is more than temporary; both groups are unable to recover from initial misinterpretations of such sentences. Children’s inability to flexibly shift between competing interpretations of ambiguous sentences may be due to their relatively immature EF [11,12]. Indeed, children with higher EF are better able to overcome initial misinterpretations of structurally-ambiguous sentences [13]; this finding is consistent with adult research showing that interventions that improve EF lead to improvements in adults’ comprehension of structurally ambiguous sentences [14].

Ambiguity is prevalent at the lexical level as well. In the sentence “There were dates and apples in the fruit bowl,” *dates* is temporarily ambiguous; it could refer to days of the month, a social activity, or a fruit. Compared to unambiguous words, lexically-ambiguous words such as homophones evoke greater activity in regions of adults’ PFC associated with EF [15,16]. This activation only occurs in sentences where the ambiguity in meaning is subsequently resolved, indicating that EF is engaged not by the presence of competing interpretations but by the process of inhibiting incorrect interpretations [17]. Consistent with this claim, children with higher EF are better at inhibiting the inappropriate meanings of homophones [18].

For sentences with either structural or lexical ambiguity, there are multiple competing interpretations. To comprehend such ambiguous sentences, both children and adults engage EF to inhibit incorrect competing interpretations. Importantly, though, EF may also be involved in everyday language use that does not involve ambiguous sentences. Recall our earlier example: a single object, such as an apple, can be identified using a variety of dimensions and words. Both children [19,20] and adults [21] incrementally process speech, anticipating the next word before it is heard. While incremental processing speeds language comprehension in general, it may also lead to incorrect predictions. Listeners will sometimes anticipate the wrong word and/or dimension, requiring them to engage EF to inhibit their attention to the irrelevant dimension and shift to the relevant dimension.

The ability to flexibly shift between dimensions when comprehending speech may be particularly difficult for children, given their relatively immature EF abilities. One clue comes from a hallmark EF task, the Dimensional Change Card Sort Task (DCCS; [22]). After sorting cards to match based on shape, children perseverate, continuing to sort based on shape, even when asked to switch and sort based on color. We hypothesize that children may face similar challenges in language comprehension. That is, children’s processing of the sentence “Where’s the apple?” may be disrupted if this sentence was preceded by a sequence of sentences labeling objects by their colors (“Where’s the blue one?”).

In the current experiment, we asked whether dimensional switches affect young children’s language comprehension. To test this hypothesis, we measured 3-year-olds’ comprehension of sentences identifying objects along a single dimension. On each trial, children saw a pair of objects, and heard speech guiding their attention to the target object via either its color or its name. Trials were arranged into two blocks. In the Pre-Switch block, each object was identified using a single dimension (e.g., always by color), and in the Post-Switch block each object was identified using a different dimension (e.g., always by name). The order of the dimensions was counterbalanced across children.

We predicted that children would be significantly less accurate in comprehending speech following the dimensional switch at the beginning of the Post-Switch block. As in the DCCS, children may perseverate, expecting objects to continue to be labeled using the same dimension. However, we also expected the decrease in accuracy during the Post-Switch block to be temporary, and confined to trials at the beginning of the block. With consistent exposure to objects identified along a new dimension, children should quickly adjust their expectations. Lastly, we predicted that the magnitude of the decrease in accuracy would be related to individual differences in EF, such that children with higher EF (i.e., those who succeed in switching dimensions during the DCCS) would be less affected by the dimensional change.

## Materials and Methods

### Participants

The final sample included fifty-six full-term children (25 female) with a mean age of 3 years and 8 months (range = 3;5 to 3;11). Parents reported that their children heard fewer than 10 hours per week of a language other than English, had normal hearing and vision, and were currently free of ear infections. Five additional participants were excluded due to technical error (n = 1), inability to code eye movements because of glare on glasses (n = 1), failure to complete all tasks (n = 1), or side bias (the child looked at a single side of the screen more than 80% of the time before target word onset; n = 2). All parents provided written informed consent and children provided oral assent. Children’s assent was documented on paper and attached to the parents’ written consent forms. All experimental protocols, including the procedures for obtaining informed consent, were approved by the University of Wisconsin-Madison IRB.

### Measure of receptive vocabulary

All children completed the Peabody Picture Vocabulary Test, Fourth Edition (PPVT-4; [23]), a norm-referenced test designed to measure receptive vocabulary. On each trial, children were shown a series of four line drawings and asked to point to the picture that best matched the meaning of a spoken word. We used age-adjusted standard scores as our measure of receptive vocabulary.

### Measure of Executive Function

Children also completed the NIH Toolbox version of the Dimensional Change Card Sort [24]. The task was administered using the Assessment Center website on a Lenovo laptop connected to a 48 cm widescreen LCD monitor. Children responded using a button box with two large red buttons.

In the NIH Toolbox version of the DCCS, children are first trained to match a series of pictures (white boats and brown rabbits) to target pictures reversing the dimensions (brown boats and white rabbits). Training consists of 4 trials matching pictures based on shape followed by 4 trials matching pictures based on color. Children receive computer-generated feedback for both correct and incorrect responses. Each set of 4 trials is repeated up to twice if children make 2 or more errors in the set. Following training, children are then asked to match a different set of pictures (blue trucks and yellow balls) to target pictures reversing the dimensions. Testing consists of 5 trials matching based on color (pre switch phase), followed by 5 trials matching based on shape (post switch phase), and 30 trials alternating between matching based on shape and color (mixed phase). Children only advance to the next phase in testing if they make fewer than 2 errors in the previous phase. Thirty-five children who perseverated on the DCCS (9 who failed the pre switch and 24 who failed post switch phase) were categorized as Low EF, while 21 children who succeeded in switching between rules (i.e., passed the post switch phase) were categorized as High EF.

Although research with adults suggests that EF is divisible into different components [3], these components may not be clearly differentiated in young children [25]. For this reason, we selected a single, omnibus measure of EF that taps multiple components of EF. We chose the DCCS as our measure of EF because of its similarity to the shifting required in our language comprehension task. We discuss the limitations of this decision in the General Discussion.

### Measure of Online Language Comprehension

Children’s real-time comprehension of familiar words was assessed using the looking-while-listening procedure [26]. On each trial, two pictures of familiar objects were displayed simultaneously in silence for 2 seconds. Children then heard speech identifying one of the objects using either the name of the object (e.g., ‘Find the sock’) or its color (e.g., ‘Find the blue one’). The pictures remained on screen for 1 second after the offset of the sound. A blank screen appeared for 1 second between trials.

#### Images

Thirty-two familiar objects were selected; images were edited using Adobe Photoshop for size and color. Images were also edited to approximately match in visual salience. Saliency match was initially determined subjectively and subsequently confirmed objectively by comparing children’s fixations to both objects *before* the onset of the target word. All objects were monochromatic, and depicted in one of eight colors familiar to children at this age. For a list of objects and colors see Table 1. Objects were presented on gray backgrounds and aligned horizontally on a 140 cm LCD television. Children sat approximately 1 m away from the screen on their caregiver’s lap. Caregivers wore opaque sunglasses to restrict their view of the images.

**Table 1:** List of familiar objects, sorted by color, that were used as stimuli.

Color	Familiar Objects			
Orange	Slide	Purse	Plate	Dress
Blue	Crib	Sock	Scissors	Glasses
Red	Shorts	Knife	Comb	Belt
Green	Boot	Bucket	Bib	Slipper
Yellow	Zipper	Shovel	Donut	Puzzle
Black	Hammer	Hose	Can	Tape
White	Flag	Necklace	Chalk	Ladder
Brown	Ladder	Lamp	Muffin	Pudding

#### Audio

Speech stimuli on each trial consisted of two sentences: a carrier phrase with the target word in the final position (e.g., ‘Find the sock’), followed by an attention getter (e.g., ‘Check that out!’). A female native speaker recorded multiple tokens of each sentence. Tokens were selected to have similar intonation contours and were edited using Praat to match duration (see Table 2) and intensity (65 dB).

**Table 2:** Normed durations of each audio segment.

**Segment**	Silence	Carrier	Target	Silence	Attention Getter	Silence
**Example**	…	Find the	sock	…	That's cool!	…
**Duration**	2000	924	986	1000	1249	1000

#### Object pairs

The 32 objects were yoked into 16 pairs such that the four target words for each yoked pair (each object’s name & color) were matched on the proportion of 30-month-olds reported to produce each word (according to the MCDI database; http://www.cdi-clex.org/). The yoked pairs were also selected to ensure that the onsets of the four target words were phonologically distinct. Each yoked pair was presented only once to each child, with one object identified using either its name or color. There were no repetitions of objects across trials. The target object and the dimension used to identify the object (name vs. color) were counterbalanced between children. This ensured that across all children, each object was the target and the distractor an equal number of times, and each object was identified using its name and its color an equal number of times.

#### Trial order

There were a total of 16 trials for each participant. Trials were arranged into 2 blocks with 8 trials in each block. In the Name block, each object was identified using its name. In the Color block, each object was identified using its color. There was no break between blocks. Assignment of each yoked object pair to the Name vs. Color block was counterbalanced between participants. Additionally, block order was counterbalanced such that half of the participants started with the Name block and the other half started with the Color block. All counterbalancing was performed separately for the two EF subgroups (children with Low EF and children with High EF). This ensured that statistical analyses comparing Low and High EF children were not confounded with counterbalanced variables (e.g., more Color to Name switches for Low EF children).

#### Eye movement coding

Children’s looking behavior during each trial was video recorded. Eye-movements were coded on a frame-by-frame basis by trained coders blind to target side and condition using custom software. On each frame, the coder indicated whether the child was looking at the picture on the left, the picture on the right, or away from the display. This yielded a record of each child’s eye movements in 33 ms increments as the sentence unfolded in real time. To determine reliability, 25% of the sessions were independently recoded. Interobserver agreement was calculated using two measures: 1) the proportion of all frames on which coders agreed on the gaze location and 2) the mean proportion of shifts in gaze on which coders agreed within one frame. Reliability was 98.9% for the first measure and 97.1% for the second measure.

#### Measures of online speech processing

Children’s accuracy was calculated as the proportion of time spent looking at the target object out of the total time spent looking at either object during a critical window 300 to 1800 ms following target word onset [26]. Eye-movements occurring either before or after this window were not considered to be stimulus-driven. Trials were excluded if the child was not fixating either object for more than 33% of the critical window (500 ms) or if the child was talking during the onset of the target word. Out of the maximum of 16 trials, on average each child contributed 14.4 trials to the accuracy analyses.

A more recent analytic approach to analyzing online speech processing uses growth curve analysis (GCA) to quantify and assess the shapes of the time course in listeners’ fixations to the target object [27]. With GCA, we can analyze changes in children’s fixations to the target object throughout the critical window, rather than collapsing across the critical window (i.e., mean accuracy). All analyses of the time course of fixations were restricted to the same critical window used for calculating mean accuracy (300 to 1800ms following target word onset). The same exclusionary criteria were used to remove trials where the child was inattentive or talking—resulting in the same number of trials per child as the accuracy analyses.

GCA with orthogonal polynomials was used to quantify fixation differences within the window of analysis. With orthogonal polynomials, the intercept is centered and reflects the average overall fixation proportion (and is thus analogous to mean accuracy). The linear time term reflects the monotonic change in fixation proportion (i.e., the average change in fixations to the target for each unit increase in time). The quadratic time term captures the rate of the symmetric rise and fall around the central inflexion point (i.e., the bowing around the peak in fixation proportions). Similarly, the cubic time term captures the steepness of the rates of rise and fall for inflections around the tails (i.e., changes in fixation proportions at the beginning and end of the window). The empirical logit transformation [28] was used to accommodate the binary nature of the data (i.e., fixations were either to the target or distractor) in a way that is robust to values near the boundaries (i.e., fixation proportions of 0 or 1). All models were fit using Maximum Likelihood Estimation and compared with a likelihood ratio test using log-likelihood (-2LL), which is distributed as χ^2^ with the degrees of freedom corresponding to the difference in the number of parameters included in each model.

The statistical analyses described below were conducted using both the more traditional (mean accuracy) and recent (GCA) measures of online language comprehension, providing an opportunity to assess the links between two related but distinct analytic approaches. Following Barr et al.’s [29] recommendation, the full random effects structures were included in each model. All analyses were carried out in RStudio (version 0.98.1056) using the lme4 package version 1.1–7.

### Procedure

Each session began with a 5-minute briefing, during which the experimenter obtained written consent from the caregiver and verbal assent from the child. When both caregiver and child were comfortable, they were seated in a soundproof booth where the child completed the DCCS task. Immediately afterwards, both caregiver and child moved to a second soundproof booth where the child completed the looking-while-listening procedure. Children were then allowed a short break to play with toys in the playroom. Following the break, both child and caregiver returned to the first soundproof booth to complete the PPVT-4. Children were then allowed to pick out two thank you gifts, while caregivers completed a demographic questionnaire and vocabulary checklist. Each session lasted approximately 1 hour.

## Results

### Mean Accuracy

**Fig 1:**
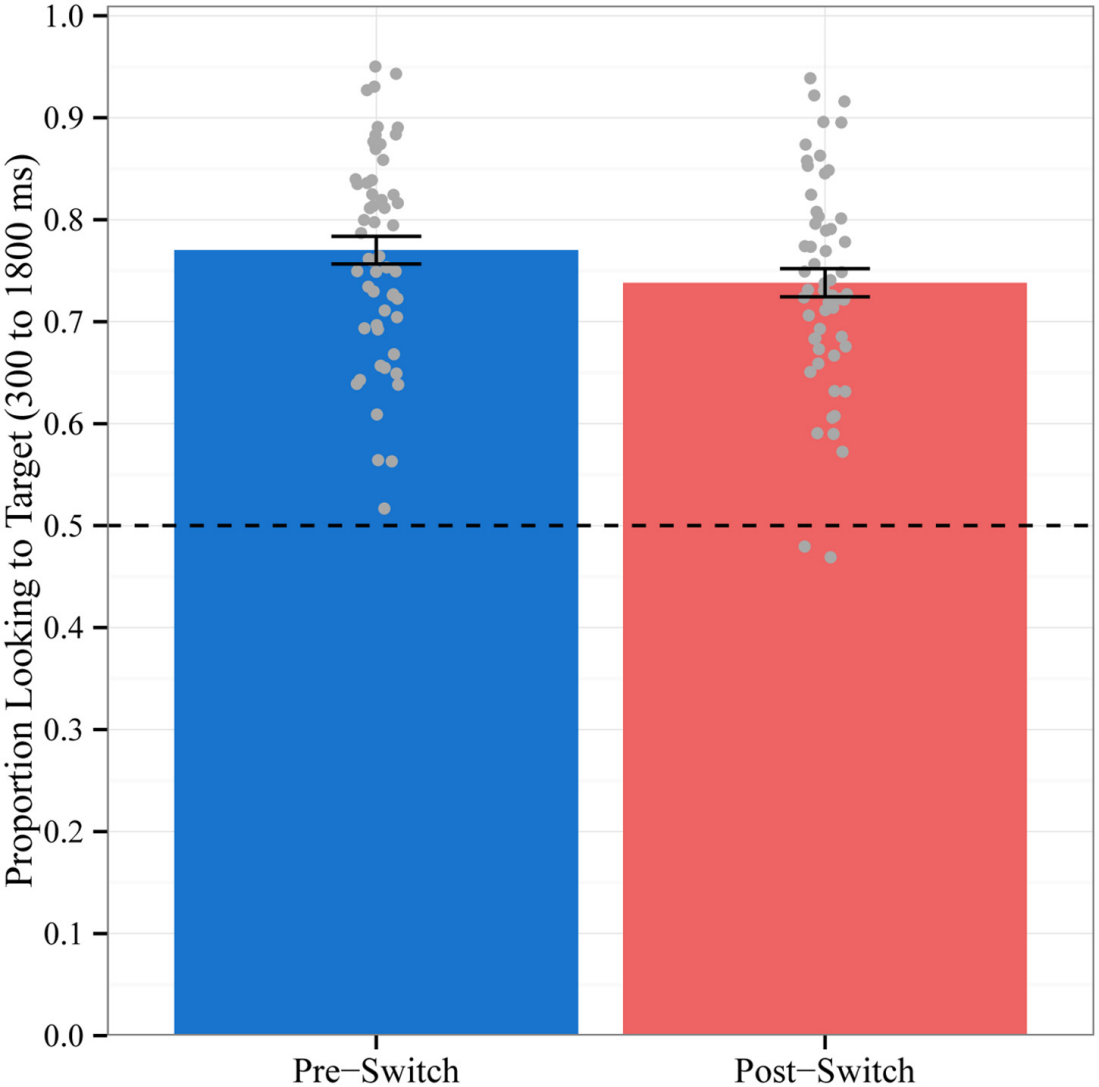
Mean Accuracy by Block. Proportion of time spent looking to the target object out of the total time spent looking at both objects during the critical window for trials before the dimensional change (Pre-Switch) and after the dimensional change (Post-Switch). Chance = 0.5. Data points represent the proportion for each subject averaged across trials. Error bars represent +/- 1 SE.

Our central hypothesis was that the dimensional switch midway through the task would impact word recognition: we predicted that children would be significantly less accurate in fixating the target object on trials following the dimensional switch (Post-Switch trials) compared to trials before the dimensional switch (Pre-Switch trials). To test this hypothesis, we estimated a linear mixed-effects model in which we regressed children’s accuracy on trial type (contrast coded as -0.5 for Pre-Switch trials and 0.5 for Post-Switch trials). We included a by-subject random intercept and a by-subject random slope for trial type. Children were significantly less accurate on Post-Switch (M = 73.8%, SD = 10.3%) compared to Pre-Switch trials (M = 77.0%, SD = 10.2%; see Fig 1). The within-subject effect of trial type was statistically significant: b = -0.03, F(1,54.3) = 4.1, p < .05. These results confirm that children’s word recognition is disrupted when objects are identified using a new, different dimension.

Our second hypothesis was that the decrease in word recognition accuracy following the dimensional switch would be transient: we predicted that children would be significantly less accurate in fixating the target object only for trials at the beginning of the Post-Switch block. To test this hypothesis, we estimated a linear mixed-effects model in which we regressed mean accuracy on trial type, which was divided into four levels: the first & second half of the Pre-Switch block and the first & second half of the Post-Switch block. Following the recommendation of Abelson and Prentice [30], we tested whether the hypothesized contrast of interest (.25, .25, -.75, .25) was statistically significant and whether the remaining two orthogonal contrasts (coded as .5, -.5, 0, 0 and .33, .33, 0, -.67) were not significant. Inspection of the residual orthogonal contrasts is necessary to determine that the pattern of results is fit only by the hypothesized pattern and not by any other systematic pattern. A by-subject random intercept and by-subject random slopes for all three contrasts were included in the random effects structure.

**Fig 2:**
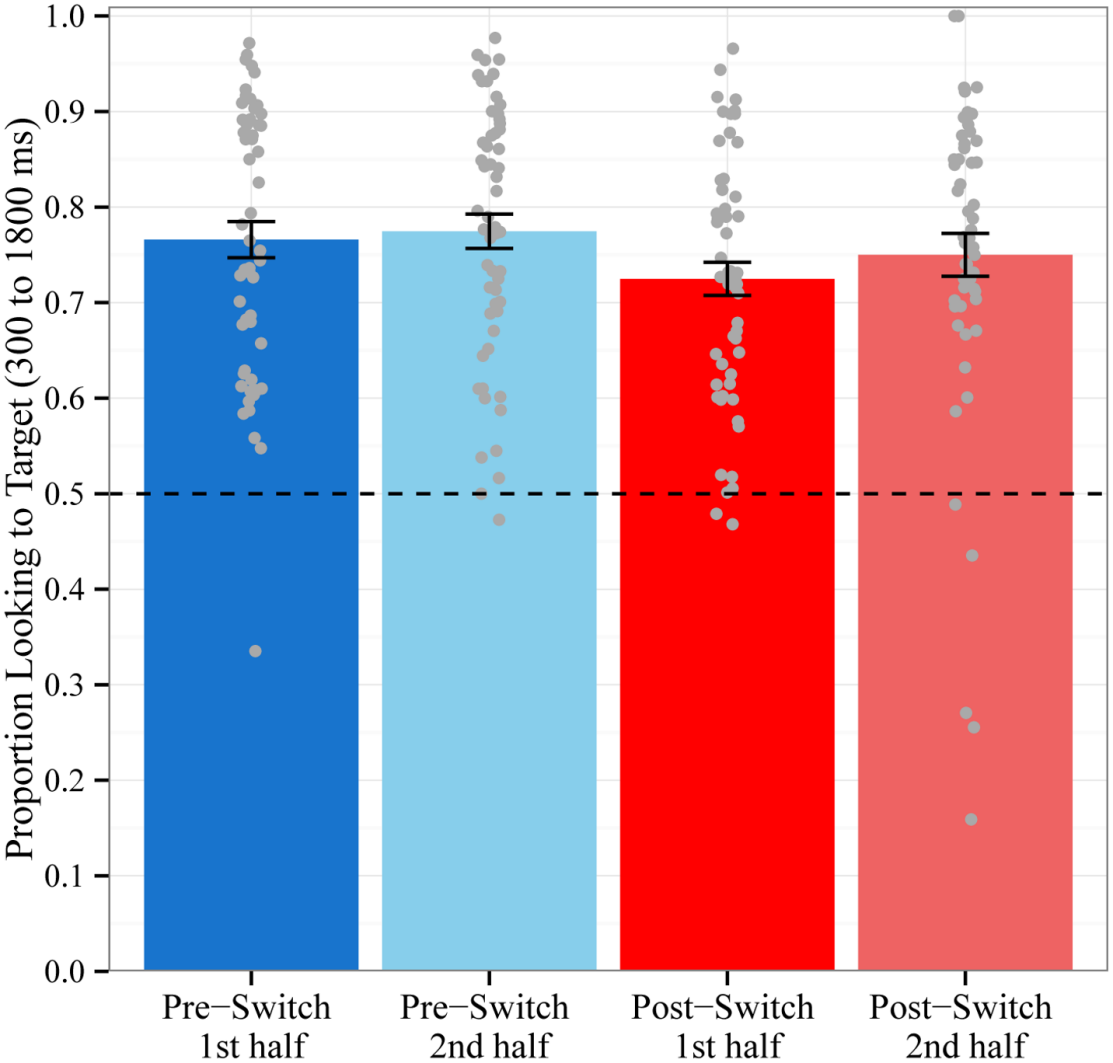
Mean Accuracy by Block Halves. Proportion of time spent looking to the target object out of the total time spent looking at both objects during the critical window for trials in the first half and second half of the Pre-Switch and Post-Switch blocks. Chance = 0.5. Data points represent the proportion for each subject averaged across trials. Error bars represent +/- 1 SE.

Consistent with the hypothesized contrast of interest, children were less accurate in fixating the target object on trials in the first half of the Post-Switch block (M = 72.5%, SD = 13.0%) compared to trials in the second half of the same block (M = 75.0%, SD = 16.8%), trials in the first half of the Pre-Switch block (M = 76.6%, SD = 14.1%), and trials in the second half of the Pre-Switch block (M = 77.5%, SD = 13.4%; see Fig 2). The contrast of interest (.25, .25, -.75, .25) was statistically significant [b = 0.05, F(1,53.7) = 6.2. p = .016], while the contrasts testing the residual trial type variance (.5, -.5, 0, 0 and .33, .33, 0, -.67) were not [F(2,53.1) .33, p = .72]. These results indicate that the decrease in children’s accuracy was indeed transient: children were significantly less accurate in fixating the target object only on trials in the first half of the Post-Switch block. This pattern of results suggests that the observed decrease in word recognition accuracy following the dimensional change was not due to fatigue or inattention towards the end of the looking-while-listening procedure, since accuracy improves again at the end of the Post-Switch block.

### Growth Curve Analysis (GCA)

**Fig 3:**
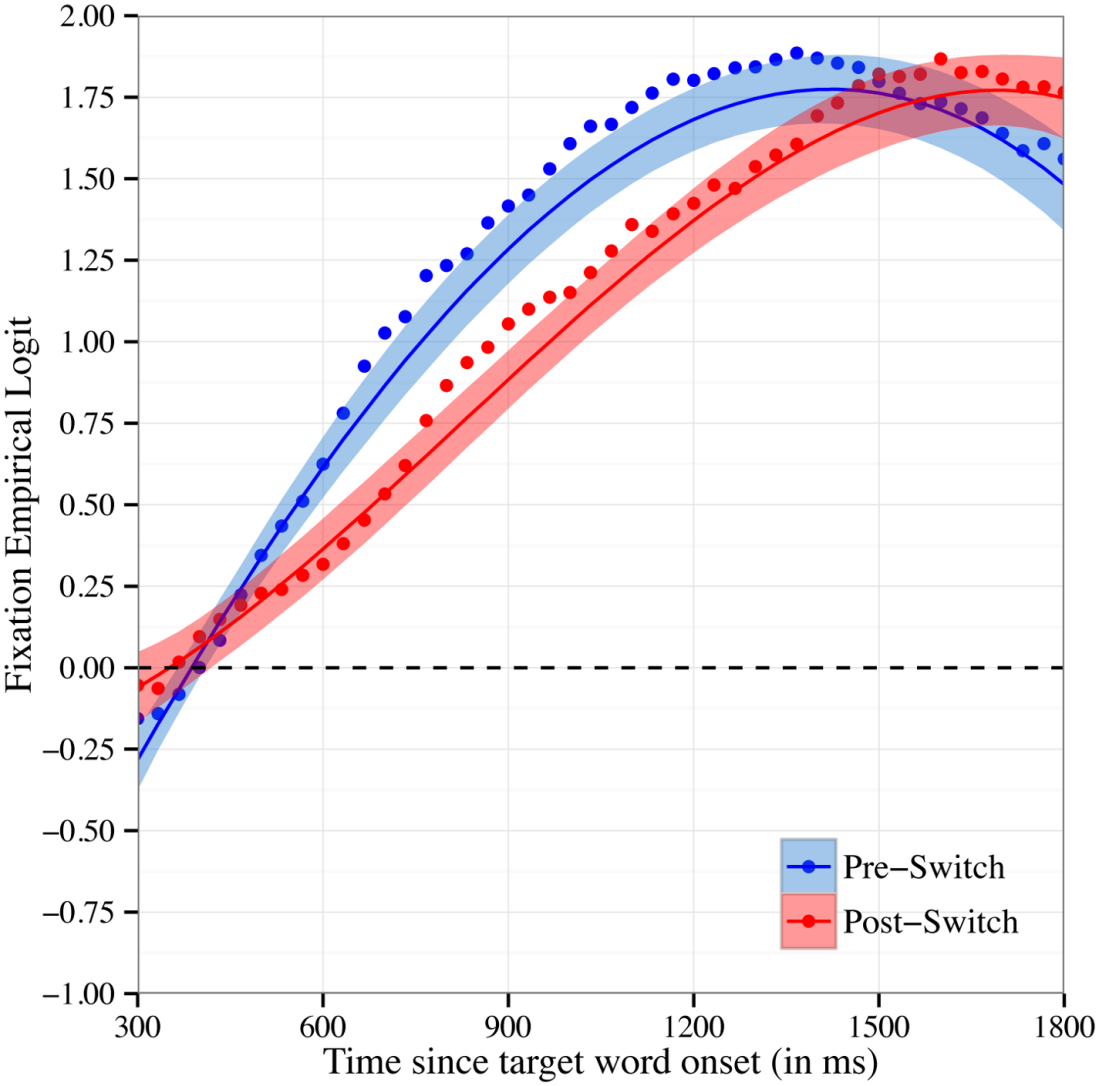
Time Course of Fixations by Block. Time course of fixations to the target object on Pre-Switch and Post-Switch trials. Fixations are plotted as the empirical log-odds averaged across participants. Chance = 0 log-odds. Data points are observed behavioral data and lines the growth curve fits (ribbons around the lines indicate +/- 1 SE).

Recall that the mean accuracy analyses reported above revealed that children were significantly less accurate in fixating the target object on Post-Switch compared to Pre-Switch trials. Fig 3 shows the time course of fixations to the target object in Pre-Switch compared to Post-Switch trials at 33ms intervals. We modeled the time course of children’s fixations with fixed effects of trial type (contrast coded as -0.5 for Pre-Switch and 0.5 for Post-Switch) on all time terms. There was a marginally significant effect of trial type on the intercept: b = -0.16, χ^2^(1) = 3.4, p < .07. Children fixated the target object at an overall lower probability for Post-Switch compared to Pre-Switch trials. These findings are consistent with the results from the mean accuracy analyses above. Moreover, there was a significant effect of trial type on quadratic time, b = 1.3, χ^2^(1) = 13.3, p < .001. This difference captures the slower increase in children’s fixations to the target object on Post-Switch trials compared to Pre-Switch trials, indicating that children’s word recognition is slowed following the dimensional shift. There was not a significant effect of trial type on either linear or cubic time (p’s > .4).

Children’s speed in speech processing is traditionally calculated as the latency (in ms) to shift from the distractor to the target image [26]. Only trials where the child is fixating the distractor at target word onset and shifts to the target within the 300 to 1800 ms window post target word onset are included in latency analyses. Out of the maximum 16 trials, on average each child contributed 5.7 trials to our latency analyses. The slower increase in fixations to the target object on Post-Switch compared to Pre-Switch trials was not due to longer latencies (in ms) to shift from the distractor to the target object at target word onset, b = 40.4, F(1,54.75) = 1.0, p = .32. This suggests that children’s slower increase in fixations to the target object on Post-Switch trials are due to changes in their looking behavior both when they are fixating the distractor and when they are fixating the target object at target word onset. For example, when fixating the target object at target word onset, an increase in looks back to the distractor object will lead to a slower increase in the proportion of fixations to the target. With few usable latency trials per subject, however, our latency estimates were potentially noisy and this negative finding should be interpreted with caution.

**Fig 4:**
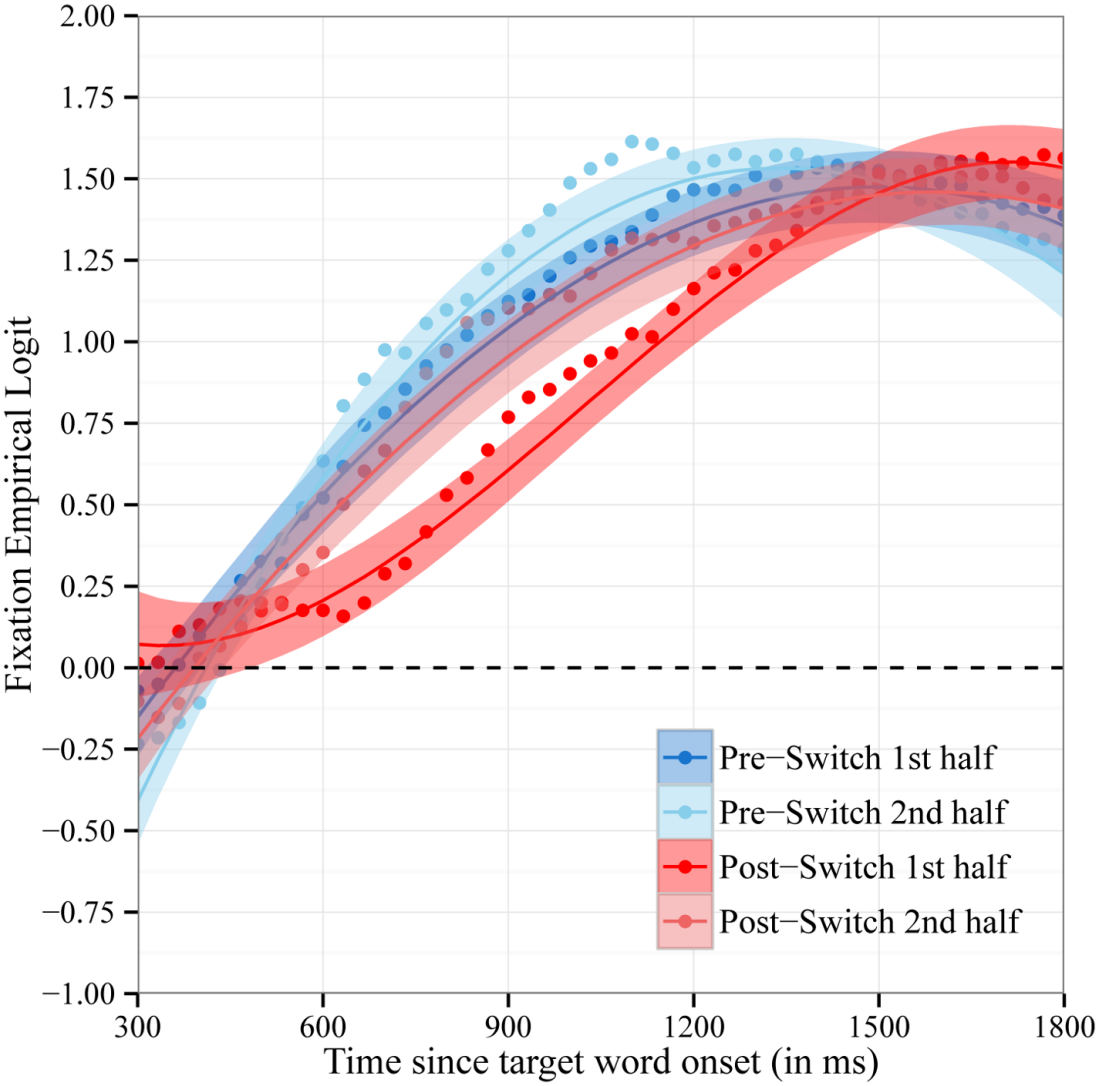
Time Course of Fixations by Block Halves. Time course of fixations to the target object on trials in the first half and second half of the Pre-Switch and Post-Switch blocks. Fixations are plotted as the empirical log-odds averaged across participants. Chance = 0 log-odds. Data points are observed behavioral data and lines the growth curve fits (ribbons around the lines indicate +/- 1 SE).

The accuracy analyses reported above revealed that the decrease in children’s accuracy in fixating the target object on Post-Switch trials was temporary, with their accuracy in fixating the target recovering in the second half of the Post-Switch trials. Fig 4 shows the time course of fixations to the target object for Pre- and Post-Switch trials with each block split into halves. We modeled the time course of children’s fixations with fixed effects of trial type on all time terms. The fixed effect of trial type used the same three contrast codes as before: the hypothesized contrast (.25, .25, -.75, .25) and two orthogonal contrasts (.5, -.5, 0, 0 and .33, .33, 0, -.67). There was a statistically significant effect of the hypothesized contrast (.25, .25, -.75, .25) on the intercept, b = 0.17, χ^2^(1) = 7.6, p < .01, and a marginally significant effect on cubic time, b = 0.5, χ^2^(1) = 3.5, p < .07. There was not a significant effect of the contrasts testing the residual between-trial variance on the intercept, χ^2^(2) = 1.0 p = .6, and cubic time, χ^2^(2) = 0.4, p = .8. This confirms that the effect of trial type on the intercept and cubic time is only fit by the hypothesized pattern. The effect of trial type on the intercept indicates that children were overall less accurate in fixating the target object on trials in the first half of the Post-Switch block (i.e., immediately following the dimensional shift). The effect of trial type on cubic time captures the delayed increase in fixations to the target object (i.e., departure from baseline) for trials in the first half of the Post-Switch block. There was a statistically significant effect of the hypothesized contrast on quadratic time (b = -1.4, χ^2^(1) = 15.0, p < .001). However, there was also a significant effect of the contrasts testing the residual between-trial variance on quadratic time, χ^2^(2) = 8.2, p < .02. This indicates that the effect of trial type on quadratic time was not fit by only the hypothesized pattern; further patterns were needed to account for the observed effect of trial type on quadratic time.

Consistent with the traditional accuracy analyses, growth curve analyses indicate that children’s accuracy in word recognition decreased following a dimensional switch in labels and that this decrease was temporary. Unlike the traditional mean accuracy analyses, however, growth curve analyses provide insight into the mechanisms underlying the decrease in word recognition accuracy. Following the dimensional switch, children’s fixations to the target object increased at a slower rate, and this increase was delayed. It is important to note that children eventually fixated the target object at equal proportions on Pre-Switch and Post-Switch trials, reaching an asymptote around 1.75 and 1.5 log-odds (the equivalent of 85 to 90% accuracy) at the end of the critical window (see Figs 3 & 4). This pattern of results indicates that following a dimensional switch, children’s word recognition is slower and less efficient but not completely impaired.

### Order Effects

The mean accuracy and GCA results above indicate that switching between dimensions disrupts children’s word recognition. The order of the two dimensions was counterbalanced across children. To test whether children experienced the same decrease when switching from Color to Name (CN) versus Name to Color (NC) we added dimension order (contrast coded as -0.5 for CN and 0.5 for NC) and its interaction with trial type as a regressor in each model.

In the mean accuracy analyses, dimension order did not moderate the effect of trial type (Pre-Switch vs. Post-Switch) on mean accuracy [F(1,53.3) = .1, p = .78], nor did it moderate the temporary effect of trial type (first half vs. second half of trials in the Pre-Switch and Post-Switch blocks) [F(1,52.7) = .02, p = .89]. In the GCA models, dimension order did not moderate the effect of trial type on the intercept [χ^2^(1) = .37, p = .5] nor did it moderate the temporary effect of trial type on the intercept [χ^2^(1) = 0, p = 1]. The pattern of results from both the mean accuracy and growth curve analyses indicates that children experienced the same overall decrease in word recognition accuracy regardless of the order of the dimensions.

Although dimension order did not moderate the effect of trial type on the intercept in the GCA models, it did moderate the effect of trial type on other time terms. The steeper rise in children’s fixations to the target on Pre-Switch compared to Post-Switch trials (i.e., the effect of trial type on quadratic time described above) was significantly greater when switching from Color to Name than when switching from Name to Color, b = -2.1, χ^2^(1) = 10.5, p = .001. Likewise, the delayed increase in fixations to the target object only for trials in the first-half of the Post-Switch block (i.e., the effect of trial type on cubic time) was significantly greater when switching from Color to Name than when switching from Name to Color, b = -1.9, χ^2^(1) = 12.1, p = .001. The residual contrasts testing the effect of trial type on cubic time were not significantly moderated by dimension order, χ^2^(2) = .66, p = .72.

Taken together, these results suggest that children’s overall accuracy in word recognition decreases when switching between dimensions regardless of the order; the slower increase in children’s fixations to the target object and the delayed departure from baseline following the dimensional switch, however, occur predominantly when switching from Color to Name. This added cost for Color to Name switching may be driven by the set of stimuli used in the current experiment. Due to children’s limited color vocabulary at this age, there were more unique objects (32) than colors (8). The repetition of colors but not names across trials could make it more difficult to shift from Colors to Names. Future studies that employ dimensions other than Color and Name will help to further elaborate whether and how specific dimensions may differentially impact children’s comprehension following a switch.

### Individual Differences in EF

We predicted that the decrease in children’s word recognition accuracy following the dimensional switch would be smaller for children with High EF: children who were better able to switch between dimensions during the DCCS sorting task would be less disrupted by the switch in dimensions during the language comprehension task. To test this hypothesis, we added EF (contrast coded as -0.5 for children with Low EF and 0.5 for children with High EF) and its interaction with trial type as a regressor in each model.

**Fig 5:**
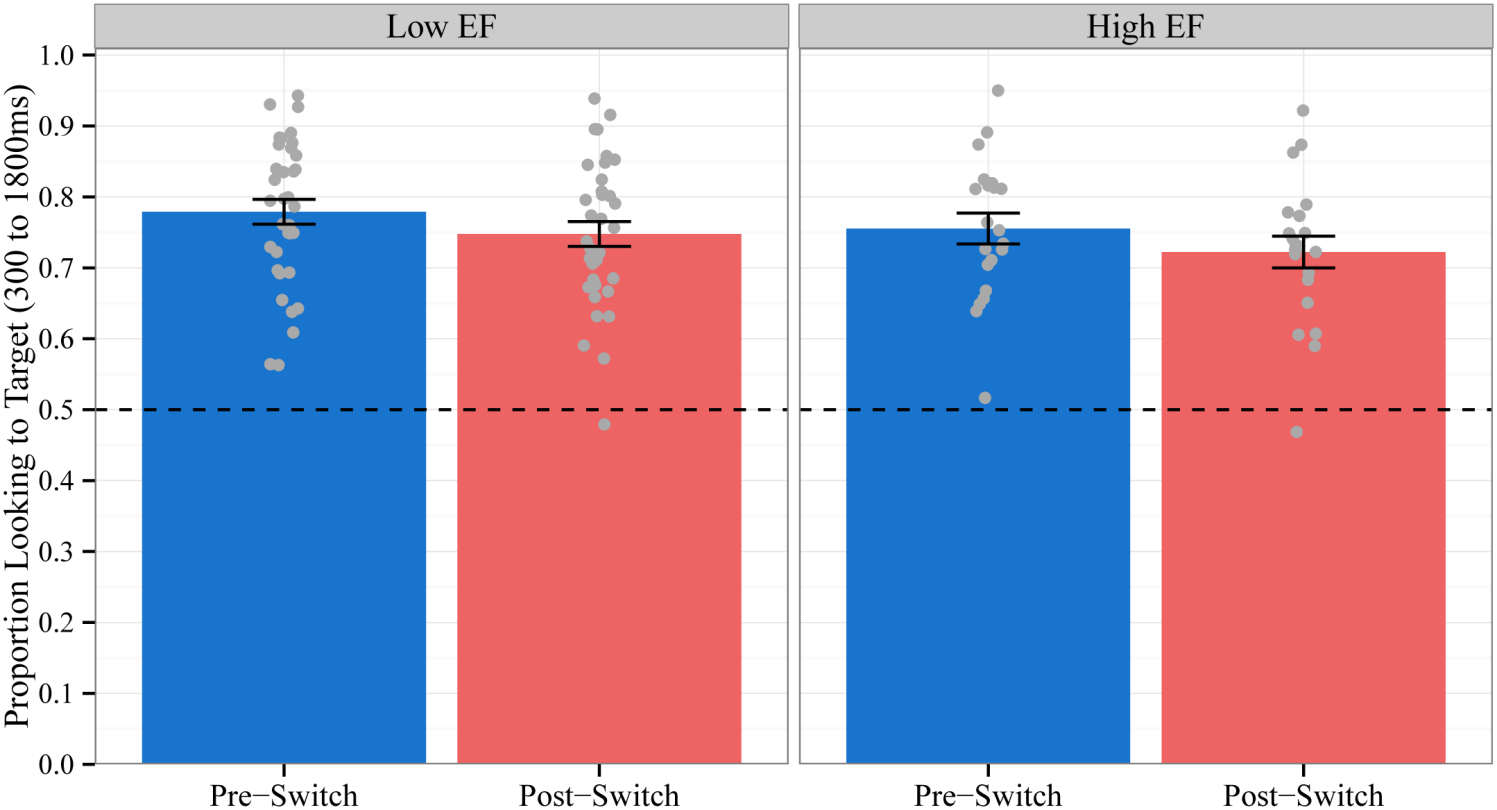
Mean Accuracy by Block by EF. Proportion of time spent looking to the target object out of the total time spent looking at both objects during the critical window for Pre-Switch and Post-Switch trials for children with low EF (left panel) compared to high EF (right panel). Chance = 0.5. Data points represent the proportion for each subject averaged across trials. Error bars represent +/- 1 SE.

**Fig 6:**
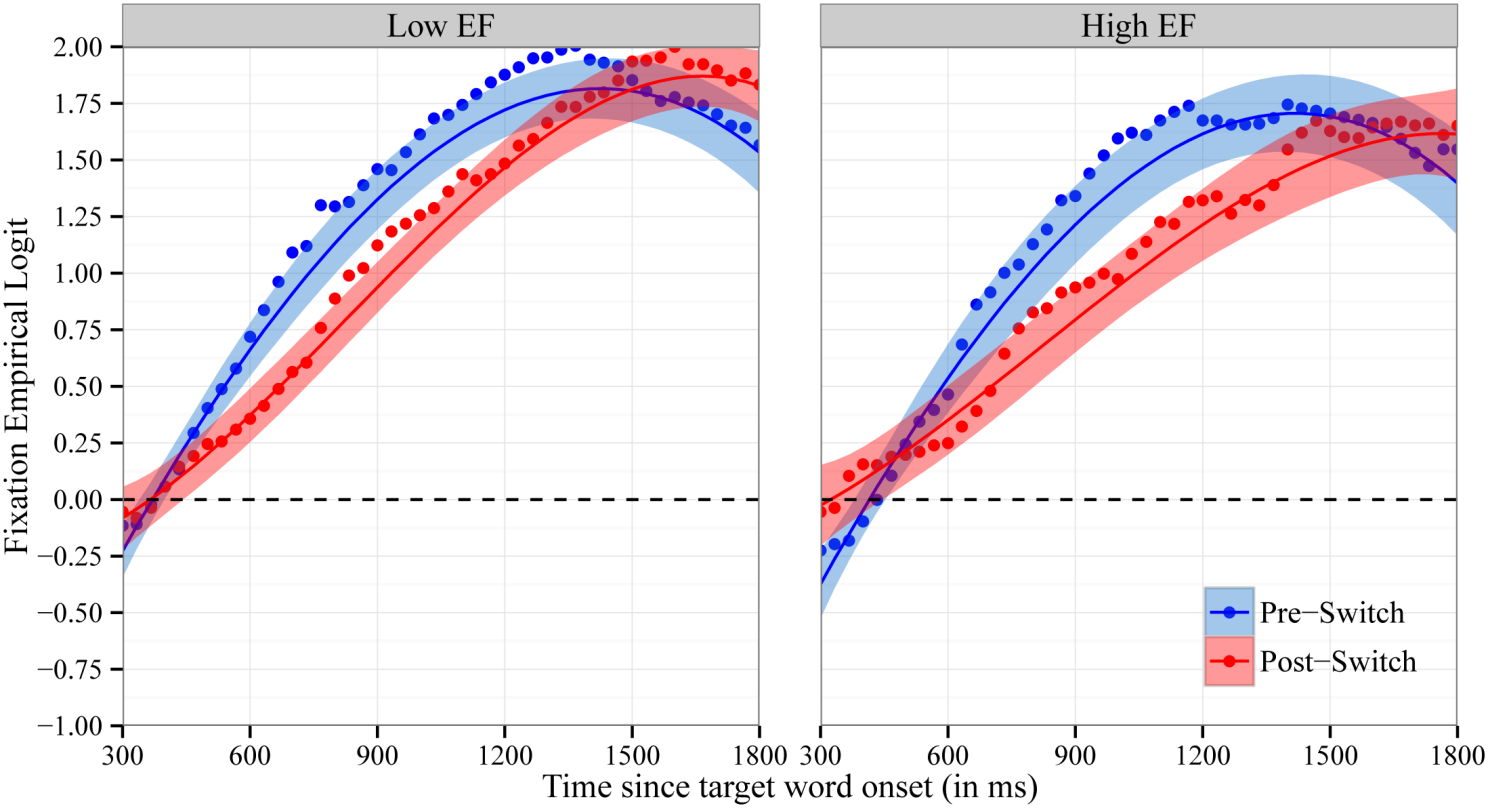
Time Course of Fixations by Block by EF. Time course of fixations to the target object on Pre-Switch and Post-Switch trials for children with low EF (left panel) compared to high EF (right panel). Fixations are plotted as the empirical log-odds averaged across participants. Chance = 0 log-odds. Data points are observed behavioral data and lines the growth curve fits (ribbons around the lines indicate +/- 1 SE).

We first examined whether EF moderated the effect of trial type (Pre-Switch vs. Post-Switch trials) on children’s accuracy in fixating the target object. Children’s mean accuracy (Fig 5) and the time course of their fixations to the target object (Fig 6) on Pre-Switch and Post-Switch trials are plotted separately for Low EF and High EF children. EF did not moderate the effect of trial type on children’s mean accuracy [F(1,53.9) = .01, p = .91], nor did EF moderate the effect of trial type on any time terms for the time course of children’s fixations (all p’s > 0.44). These results indicate that Low EF and High EF children were equally affected by the dimensional change in our language comprehension task.

**Fig 7:**
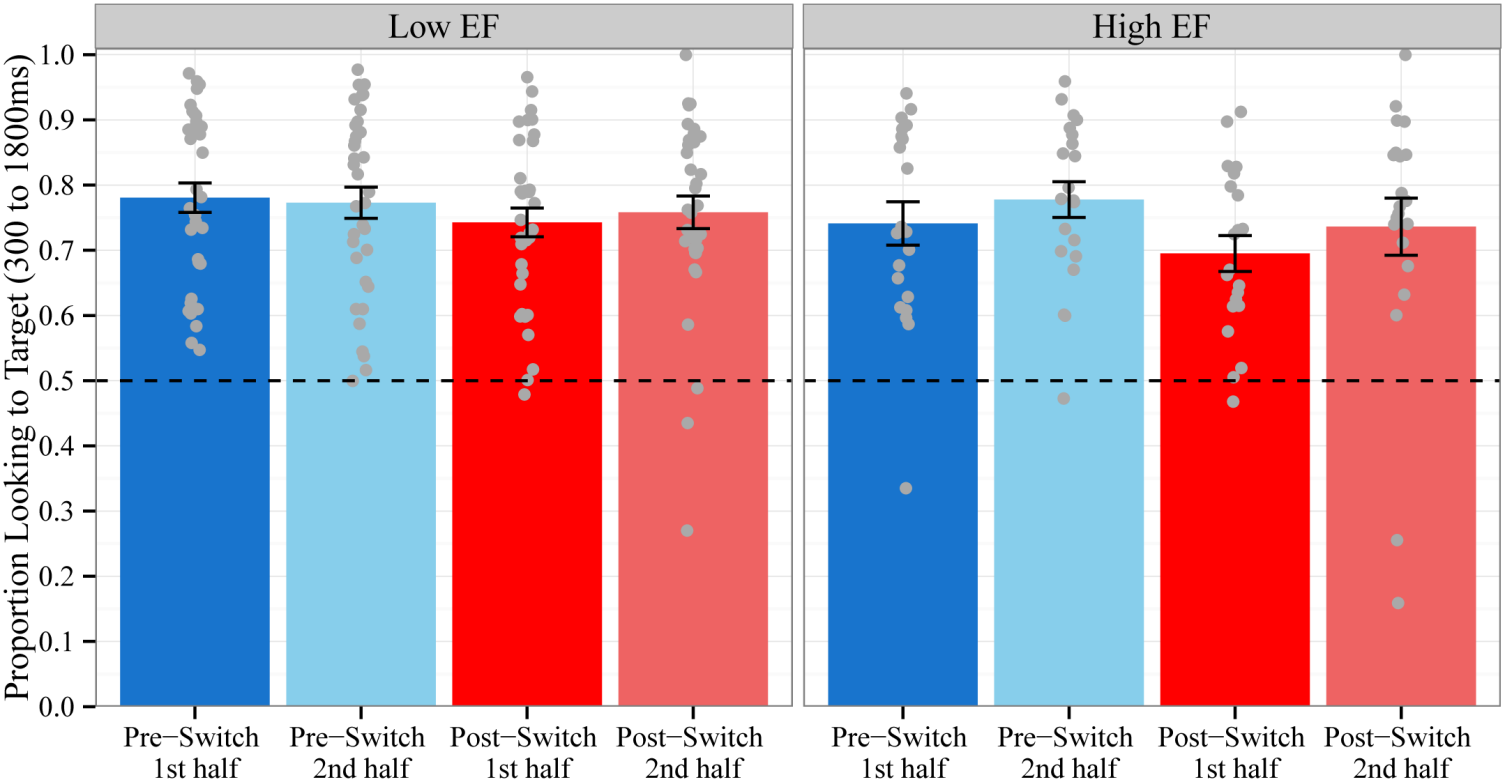
Mean Accuracy by Block Halves by EF. Proportion of time spent looking to the target object out of the total time spent looking at both objects during the critical window for trials in the first and second half of the Pre-Switch and Post-Switch blocks. Children with low EF are plotted in the left panel and high EF in the right panel. Chance = 0.5. Data points represent the proportion for each subject averaged across trials. Error bars represent +/- 1 SE.

**Fig 8:**
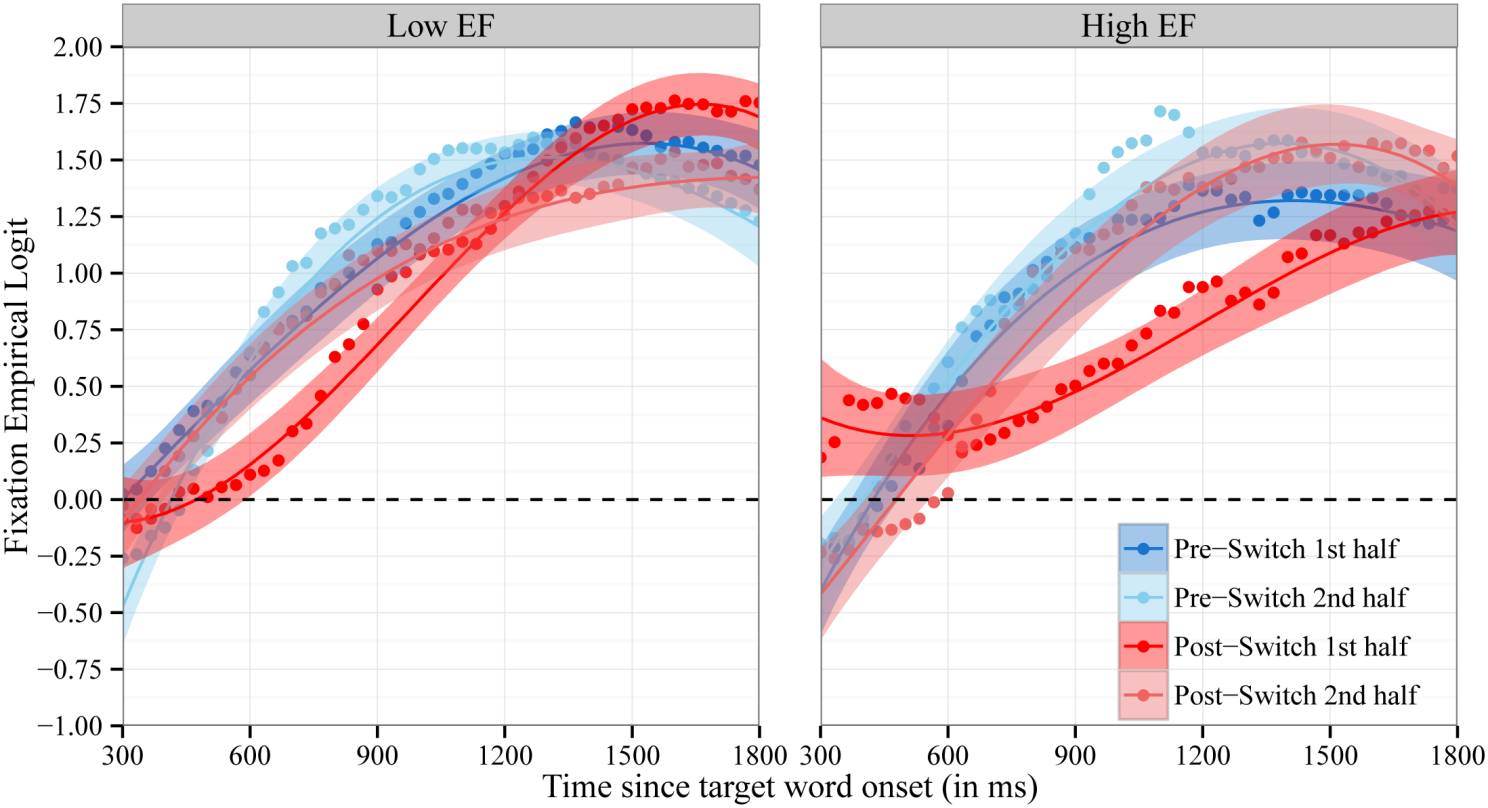
Time Course of Fixations by Block Halves by EF. Time course of fixations to the target object on trials in the first and second half of the Pre-Switch and Post-Switch blocks. Children with low EF are plotted in the left panel and children with high EF in the right panel. Fixations are plotted as the empirical log-odds averaged across participants. Chance = 0 log-odds. Data points are observed behavioral data and lines the growth curve fits (ribbons around the lines indicate +/- 1 SE).

We next examined whether EF moderated the *temporary* effect of trial type (first half vs. second half of trials in the Pre-Switch and Post-Switch blocks) on children’s accuracy in fixating the target object. Children’s mean accuracy (Fig 7) and the time course of their fixations to the target object (Fig 8) on trials in the first half compared to second half of the Pre-Switch and Post-Switch block are plotted separately for Low EF and High EF children. EF did not moderate the effect of the hypothesized contrast (.25, .25, -.75, .25) on children’s mean accuracy: F(1,53.3) = 1, p = .32. For the time course of fixations, EF significantly moderated the effect of the hypothesized contrast for trial type on linear time: b = 2.5, χ^2^(1) = 5.8, p < .02. EF did not moderate the effect of the contrasts testing the residual trial type variance on linear time: χ^2^(2) = 1.4, p = .5. This pattern of results indicates that the magnitude of the decrease in linear slope (i.e., the monotonic increase in fixations to the target object over time) for trials immediately after the dimensional switch was significantly *larger* for High EF children compared to Low EF children. This suggests that High EF children were more adversely impacted by the dimensional change in the language comprehension task and is in the opposite direction of our predictions. These results should be interpreted with caution, however, because they are likely driven by baseline differences in fixations at the start of the window (see Fig 8), with High EF children’s fixations to the target object starting above chance levels. We discuss potential reasons for the lack of an observed relationship between children’s EF and language comprehension in the General Discussion.

### Individual Differences in Receptive Vocabulary

Previous research has found that children’s online language comprehension is correlated with vocabulary: children with larger receptive vocabularies are typically more accurate in fixating the target object in a looking-while-listening task [31]. In the current experiment, however, children’s receptive vocabulary (mean-centered) was not correlated with their mean accuracy in fixating the target: b = .001, F(1,53.0) = 1.9, p = .17. Vocabulary did not moderate the effect of trial type (Pre-Switch vs. Post-Switch) on mean accuracy: F(1,52.3) = 0.23, p = 0.63. Additionally, vocabulary did not moderate the effect of trial type on any time terms for the time course of children’s fixations (all p’s > 0.25). Vocabulary also did not moderate the *temporary* effect of trial type (first half vs. second half of trials in the Pre-Switch and Post-Switch block) on mean accuracy: F(1,50.7) = .01, p = .94. Nor did vocabulary moderate the temporary effect of trial type on any time terms for the time course of fixations (all p’s > 0.20). Using children’s raw PPVT score did not change the pattern of results. We had no a priori predictions about whether vocabulary would influence children’s ability to switch between dimensions when comprehending speech. We had anticipated, however, that we would replicate the relationship between vocabulary and language comprehension that has been observed in previous research. One possible explanation was that our sample of children scored high on the PPVT (mean standard score M = 119.9, SD = 13.9); a homogenous distribution of vocabulary scores limits our ability to test whether individual differences in vocabulary predict different outcomes.

Finally, previous research has found that young children’s receptive vocabulary is strongly related to EF [32]. In the current experiment, however, children’s receptive vocabulary was not related to EF. There was no difference in receptive vocabulary [b = 4.8, t(54) = 1.3, p = .21] between children in the High EF group (M = 122.7, SD = 11.9) and the Low EF group (M = 117.9, SD = 14.6). Although the same tasks (PPVT-4 and the NIH Toolbox version of the DCCS) were used in both the current experiment and previous research [32], children in the current experiment were recruited from a narrower age range (3-year-olds) than in previous research (3- to 6-year-olds) [32].

## General Discussion

Language is used to identify objects in many different ways: by their names, by their attributes, and by the categories in which they are members. Language comprehenders must cope with this variability. Based on their language experience, listeners may generate expectations about which forms are most likely to occur. If this is the case, then a shift in the way an object is identified may have repercussions for online language comprehension. In the current experiment, we manipulated the linguistic cues in a word recognition task. During the Pre-Switch block, objects were always identified using either their name or their color. Then, in the Post-Switch block, the labeling dimension was abruptly switched.

We predicted that flexibly shifting between dimensions when comprehending speech would be difficult for 3-year-olds, who have relatively immature EF. Indeed, children’s accuracy in fixating the target object decreased in the Post-Switch block, when objects were identified using a new, different dimension. Moreover, this decrease in children’s accuracy was temporary, only occurring for trials immediately after the dimensional switch. Growth curve analyses of the time course of children’s fixations indicate that the decrease in children’s accuracy following the dimensional switch was driven by a slower increase in fixations to the target.

These findings provide the first evidence that flexibly shifting between dimensions when comprehending speech is difficult for young children. This difficulty in flexibly shifting between dimensions is consistent with the difficulty that older children experience when flexibly shifting between competing interpretations of ambiguous sentences [9] or words [18]. Shifting between dimensions, however, is not the same as shifting between different interpretations of ambiguous language. With ambiguous language, different interpretations are mutually exclusive (e.g., *on the towel* either specifies which apple or where to put the apple). In contrast, the different dimensions that can be used to identify an object are not mutually exclusive (e.g., an apple is simultaneously red, a fruit, and edible). Our findings suggest that children struggle to flexibly shift when comprehending language even in the absence of conflict.

Our findings are also in line with decades of research indicating that young children fail to switch between dimensions (shape vs. color) in the Dimensional Change Card Sort (DCCS) task [e.g., 22]. The DCCS literature suggests multiple different, but not mutually exclusive, hypotheses concerning the mechanisms underlying young children’s difficulty with dimensional changes. Perseveration in sorting may be due to an inability to represent higher-order rules [22,33]. Other accounts point to an inability to inhibit attention to the previously relevant dimension [34] or stronger active memory representations of the previously relevant dimension [35,36]. These later hypotheses provide potential insight into children’s behavior in the current experiment. Following multiple trials on which both objects and colors varied, but only one dimension was relevant (e.g., objects were only named), children may selectively attend to only the relevant dimension. After the dimensional switch, children’s word recognition may be disrupted if they are unable to inhibit their attention to the now irrelevant (but previously relevant) dimension. Conversely, children’s lack of attention to the now relevant (but previously irrelevant) dimension may result in weaker encoding and memory representation of that dimension at the onset of the target word (e.g., remembering that the other object was a sock but not remembering that it was blue). Both types of mechanisms could contribute to the slower word recognition following a dimensional change observed in our experiment.

Previous research indicates that children engage EF when comprehending and producing speech. Children with higher EF are better able to overcome initial misinterpretations of structurally ambiguous sentences [13], suppress the inappropriate meaning of homophones [18], and inhibit overgeneralization errors when producing irregular past tense forms (e.g., *flied*; [37]). We predicted that children with High EF (i.e., those who were able to switch between dimensions on the DCCS) would be better able to shift between dimensions when comprehending speech. However, individual differences in EF did *not* moderate the decrease in children’s comprehension following the dimensional switch. While the EF and language comprehension tasks were deliberately chosen to be similar (involving dimensional switches between names and colors) they differ in several important aspects. One way in which the two tasks differ is the presence of conflict. In the DCCS, different dimensions lead to mutually exclusive responses: the blue truck is sorted in one pile for the shape game (i.e., with the yellow trucks) and in another pile for the color game (i.e., with the blue balls). In our language comprehension task, however, different dimensions do *not* lead to different responses: both *blue* and *truck* identify the same object. Recent research suggests that switching between dimensions in the presence of conflict requires working memory, while switching between dimensions without conflict requires inhibition [38]. Another important way in which the two tasks differ is in their response requirements. In the DCCS, children must manually select where to sort a card, whereas in our language comprehension measure, children look at an object after it is labeled. Developmental research on children’s knowledge of physics [39] and theory of mind [40] has revealed a dissociation between children’s performance on elicited-response tasks (like the DCCS) and infants’ performance on spontaneous-response tasks (like the looking-while-listening procedure). While ostensibly similar, the dimensional change in both tasks may tap different aspects of EF or cognition due to differences in the presence of conflict and response requirements; these differences may underlie the lack of an observed relationship between EF and language comprehension in the current experiment.

Negative findings, however, are difficult to interpret, and the lack of observed relationship between EF and language comprehension may instead be due to limitations of the current methodology. Although validated for children 3 years of age and up, the NIH Toolbox version of the DCCS may be too difficult for children under the age of 7 [41]. Another limitation of the DCCS, as well as other measures of EF for children in our age range [42], is that EF is coded in a dichotomous, all-or-none fashion: children either succeed or fail on every trial. The lack of a continuous measure precludes deeper and more powerful analyses of EF. Recent work, has begun to assess elicited-response tasks like the DCCS using spontaneous-response measures like eye movements [43] and ERPs [44]. Finally, the DCCS provides an omnibus measure of EF. In future research, we will recruit older children to complete a modified version of our language comprehension task as well as a battery of EF tasks. This will allow us to better examine whether individual differences in EF are related to children’s language comprehension and if this relationship is limited to specific components of EF.

Two additional aspects of the dimensional change used in the current experiment merit discussion. First, we intentionally included only objects that do not have prototypical colors (e.g., socks). Thus, color was completely irrelevant on trials on which children are asked to identify an object using its name. However, for objects with prototypical colors, like strawberries or bananas, color is relevant even when objects are identified using their names [45]. If we had used objects with prototypical colors in the current task, children’s accuracy in fixating the target object might not have been disrupted by a switch from object names to colors. Under such circumstances, children may not need to shift their attention at all because they attend to color when identifying prototypically colored objects by name. By systematically manipulating objects’ properties (e.g., whether or not they have prototypical colors), future work can identify in which contexts a dimensional change disrupts language comprehension.

Second, switching between labeling dimensions (e.g., name vs. color) necessitates changing syntactic structures. An object’s name is a noun, its color an adjective, and an associated action a verb. Thus, in the current study, the dimensional shift was accompanied by a syntactic shift. It is therefore not possible to identify the unique contributions of the change in labeling dimensions independent of the change in labeling syntax on language comprehension. Future research will manipulate the dimensions along which objects are identified to determine the degree to which comprehension is impacted by a syntactic change (e.g., syntactic priming effects) as opposed to a dimensional change.

The results from the current experiment demonstrate that at an age in which children struggle to switch between dimensions in a sorting task, they also struggle to switch between dimensions when comprehending language. These results have consequences for other aspects of children’s language acquisition and processing. Most word learning experiments focus on how children learn the names of objects. Word learning, however, does not end there. To approximate adults’ knowledge of apples, children must also learn that they are (often) red, are fruits, can be eaten, etc. This extended word learning may be particularly challenging for young children, given their relatively immature EF. Between the ages of 3 and 6 children make marked improvements in both EF and language acquisition. These parallel improvements may be linked in important ways, such that children’s ability to control their attention influences both their language comprehension and acquisition.

## Supporting Information

S1 Appendix Data from the Original Study. (TXT)
